# Overcoming Ceftaroline Resistance in MRSA Using Ceftaroline–Carbapenem Combination Therapy

**DOI:** 10.1093/ofid/ofag009

**Published:** 2026-01-20

**Authors:** Joshua Olson, Valliammai Alaguvel, Gabriel Pérez-Parra, Allen Jankeel, Anuj K Khetarpal, Valeria Rodríguez-Guevara, Vanessa Vu, George Sakoulas, Erlinda R Ulloa

**Affiliations:** Department of Pediatrics, University of California Irvine School of Medicine, Irvine, California, USA; Department of Pediatrics, University of California Irvine School of Medicine, Irvine, California, USA; Department of Pediatrics, University of California Irvine School of Medicine, Irvine, California, USA; Department of Pediatrics, University of California Irvine School of Medicine, Irvine, California, USA; Department of Pediatrics, University of California Irvine School of Medicine, Irvine, California, USA; Department of Pediatrics, University of California Irvine School of Medicine, Irvine, California, USA; Department of Pediatrics, University of California Irvine School of Medicine, Irvine, California, USA; Division of Biology and Medicine, Brown University, Providence, Rhode Island, USA; Collaborative to Halt Antibiotic-Resistant Microbes (CHARM), Department of Pediatrics, University of California San Diego School of Medicine, La Jolla, California, USA; Division of Infectious Diseases, Sharp Rees-Stealy Medical Group, San Diego, California, USA; Department of Pediatrics, University of California Irvine School of Medicine, Irvine, California, USA; Division of Infectious Disease, Children's Hospital of Orange County, Orange, California, USA

**Keywords:** bacteremia, carbapenems, ceftaroline, ceftaroline-resistant MRSA, combination therapy

## Abstract

The limited but rising threat of ceftaroline-resistant MRSA poses a therapeutic challenge. We show that ceftaroline plus carbapenems restores activity against a resistant strain both in vitro and in a murine bacteremia model. These findings support combination therapy as a potential strategy for difficult MRSA infections, warranting further clinical investigation.

Methicillin-resistant *Staphylococcus aureus* (MRSA) remains a leading cause of persistent bacteremia and endocarditis [1]. Current first-line therapies—including vancomycin and daptomycin—often prove suboptimal in these high-burden or biofilm-associated infections due to limited bactericidal activity, emerging tolerance, or toxicity [2–5].

Ceftaroline, a fifth-generation cephalosporin, retains β-lactam activity against MRSA by targeting penicillin-binding protein 2a (PBP2a) and has gained traction for difficult infections due to its favorable safety profile [6–8]. This has raised hopes of narrowing the outcome gap between MRSA and MSSA. Yet, the slow emergence of ceftaroline resistance in MRSA, driven largely by mecA and PBP mutations, threatens one of the few β-lactams active against this pathogen [9, 10].

We have shown that combining ceftaroline with carbapenems (ertapenem or meropenem) enhances antimicrobial activity against MRSA, suppresses resistance and virulence gene expression, and augments immune-mediated killing [11]. These findings suggest a promising approach to overcome ceftaroline resistance in challenging infections. However, strategies to re-establish activity specifically against ceftaroline-resistant MRSA remain underexplored. Here, we conducted a proof-of-concept evaluation of ceftaroline–carbapenem therapy against a ceftaroline-resistant MRSA isolate both in vitro and in a murine bacteremia model to assess its potential to restore efficacy against this evolving resistance.

## METHODS

### Institutional Approval

Studies were approved by the University of California Irvine (UCI) Institutional Animal Care and Use Committee.

### Antibiotics

Antibiotics were purchased from the UCI Medical Center Pharmacy. We used the active form of ceftaroline (MedChemExpress) for in vitro studies and the prodrug ceftaroline fosamil for murine studies. Antibiotic stock solutions were prepared in molecular-grade water (Corning Cellgro) and stored at −20°C.

### Bacterial Strains and In Vitro Susceptibility Tests

All experiments were conducted using ceftaroline-resistant MRSA isolate (CDC AR-0703, sequence accession #SAMN11953853). Bacteria were grown overnight in Todd-Hewitt Broth (Hardy Diagnostics) and stored with 40% glycerol at −80°C. Fresh colonies were streaked onto Todd-Hewitt agar (THA; Hardy Diagnostics) plates each week for all experiments. Broth microdilution antimicrobial susceptibility testing was conducted under both standard (10^5^ colony-forming units [CFU]/mL) and high (10^7^ CFU/mL) inoculum conditions using cation-adjusted Mueller–Hinton broth (CA-MHB; Difco) based on Clinical and Laboratory Standards Institute (CLSI) guidelines [12]. Checkerboard assays were similarly performed in CA-MHB to assess antibiotic interactions, as defined by fractional inhibitory concentration indices (FICIs) as follows: synergy, FICI of ≤0.50; additivity, FICI of >0.50 to ≤1.0; no interaction (indifference), FICI of >1 to ≤4; antagonism, FICI of >4 [13].

### Time-Kill Assays

Kill curve assays were performed as previously described [11, 14, 15]. Bacteria were incubated with shaking at 37°C, with or without subtherapeutic ceftaroline ± ertapenem or meropenem, in either CA-MHB or Roswell Park Memorial Institute (RPMI) physiological cell culture media supplemented with 5% Luria–Bertani (LB) broth in 96-well plates. Meropenem (49 mg/L, representing peak serum concentration) [6] and ertapenem (50 mg/L) were used at sub-MIC concentrations (MIC 128 mg/L for both agents). Aliquots were collected at 6 and 24 hours and serially diluted in PBS for CFU enumeration. Synergy was defined as a ≥ 2 log_10_ CFU/mL reduction of the combination over the most active single agent and a ≥ 1 log_10_ CFU/mL reduction from baseline.

### Murine Bacteremia Model

Murine studies were performed as previously described [11]. Bacteria were injected intravenously (4.5 × 10^9^ CFU/mL) via retro-orbital vein into outbred female CD1 mice (8 to 10 weeks old, Charles River Laboratories). Two hours after infection, intraperitoneal doses (100 µL) of either PBS (control) or antibiotics were administered: ceftaroline (12 mg/kg every 8 hours) and/or ertapenem (100 mg/kg every 8 hours) [16]. Mice were euthanized with CO_2_ 26 hours after infection, followed by cervical dislocation. The kidneys were then harvested for CFU enumeration, weighed and placed in a 2-mL sterile microtube (Sarstedt) containing 1 mL of PBS and 1-mm-diameter silica beads (Biospec). They were subsequently homogenized by shaking twice at 6000 rpm for 60 seconds, using a MagNA Lyser (Roche). Aliquots from each tube were serially diluted in PBS for CFU enumeration on THA plates.

## RESULTS

### In Vitro Antibiotic Activities and Interactions Against Ceftaroline-Resistant MRSA

In vitro antibiotic activities were assessed at both standard (5 × 10^5^ CFU/mL) and high (2 × 10^7^ CFU/mL) bacterial inocula to mimic conditions relevant to infections like endocarditis. The in vitro activities of vancomycin, ceftaroline, ertapenem, and meropenem against ceftaroline-resistant MRSA AR-0703 are shown in Table 1. Full MIC results are provided in Supplementary Table 1. No clinically significant inoculum effect was observed with any of the antibiotics tested. The isolate was susceptible to vancomycin (MIC 0.5–2 mg/L; CLSI breakpoints: susceptible ≤2 mg/L, intermediate 4–8 mg/L, and resistant ≥16 mg/L) and, as expected, resistant to ceftaroline (MIC 16 mg/L; CLSI breakpoints: susceptible ≤1 mg/L, susceptible dose-dependent 2–4 mg/L, resistant ≥8 mg/L) irrespective of inoculum. Although clinical breakpoints for ertapenem and meropenem against MRSA are not established due to their lack of accepted activity [6, 17], both antibiotics exhibited high MICs. Despite this, adjunctive carbapenems restored activity against the ceftaroline-resistant isolate (AR-0703), with checkerboard assays revealing synergy between ceftaroline and either ertapenem or meropenem, regardless of bacterial density (Table 1).

**Table 1.** In Vitro Studies Under Standard (10^5^) and High (10^7^) Inocula in Cation-adjusted Mueller–Hinton Broth for Vancomycin, Ceftaroline, Ertapenem, and Meropenem Across the Methicillin-resistant *S. aureus* Isolate (AR-0703) Used in This Study

MIC (mg/L)								Checkerboard (FICI)			
Vancomycin		Ceftaroline		Ertapenem		Meropenem		Ceftaroline + Ertapenem		Ceftaroline + Meropenem	
10^5^	10^7^	10^5^	10^7^	10^5^	10^7^	10^5^	10^7^	10^5^	10^7^	10^5^	10^7^
0.50	1–2	16	16	128	256	128	128	0.50 (S)	0.50 (S)	0.50 (S)	0.50 (S)

Abbreviations: MIC, minimum inhibitory concentration; FICI, fractional inhibitory concentration index; S, synergy.

FICIs were interpreted as follows: synergy, FICI of ≤0.50; additivity, FICI of >0.50 to ≤1.0; no interaction (indifference), FICI of >1 to ≤4; antagonism, FICI of >4.

**Figure 1.**
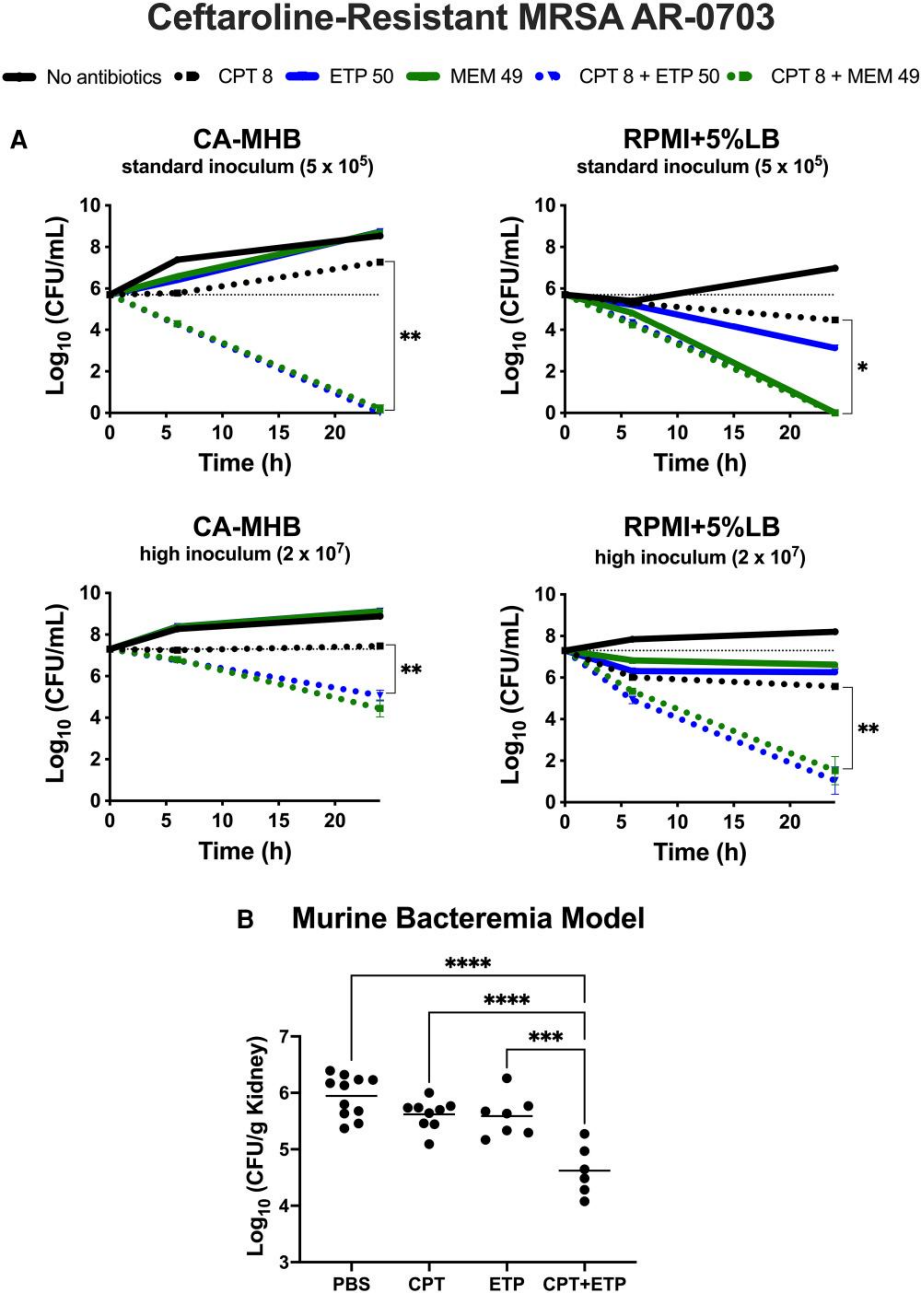
Synergistic effects of ceftaroline–carbapenem combinations against methicillin-resistant *Staphylococcus aureus* (MRSA AR-0703) under various conditions. *A*, Kill curves over 24 h demonstrating the effect of sub-MIC ceftaroline (CPT, 8 mg/L, 1/2 MIC) and carbapenems (ertapenem [ETP, 50 mg/L] or meropenem [MEM, 49 mg/L]), alone or in combination, against ceftaroline-resistant MRSA AR-0703. The MIC of ceftaroline for this isolate was 16 mg/L (CLSI breakpoints: susceptible ≤1 mg/L, susceptible dose-dependent 2–4 mg/L, resistant ≥8 mg/L). Experiments were conducted under standard (5 × 10^5^ CFU/mL) and high (2 × 10^7^ CFU/mL) inoculum conditions in CA-MHB or RPMI + 5%LB media. Combination therapy with either carbapenem showed enhanced bacterial killing compared with ceftaroline monotherapy, particularly under high inoculum, physiological (RPMI + 5%LB) conditions. Meropenem monotherapy showed comparable activity to combination therapy but only under standard inoculum conditions in RPMI + 5%LB media. *B*, Efficacy of adjunctive ertapenem therapy in a murine bacteremia model. Bacterial counts from kidneys (CFU/g) after 24 h of treatment with ceftaroline (12 mg/kg q8h) or ertapenem (100 mg/kg q8h), alone or in combination, versus no antibiotics (PBS control) are shown. Combination therapy with ceftaroline (CPT) and ertapenem (ETP) significantly reduced recoverable ceftaroline-resistant MRSA from kidneys compared with both monotherapies and the PBS control (*n* > 5). Statistical significance was determined by unpaired two-tailed *t*-test (*A*) and one-way ANOVA with multiple comparisons (*B*). **P ≤ .01, ***P ≤ .001, ****P ≤ .0001. Abbreviations: CA-MHB, cation-adjusted Mueller–Hinton broth; RPMI + 5%LB, Roswell Park Memorial Institute physiological cell culture media supplemented with 5% Luria–Bertani; CFU, colony-forming units; PBS, phosphate-buffered saline; CLSI, Clinical and Laboratory Standards Institute.

Next, we assessed the antimicrobial effectiveness of the combination through in vitro time-kill assays, conducted under bacteriologic (CA-MHB) and physiological (RPMI + 5%LB) conditions. The latter has been shown to better simulate the in vivo environment by more closely mimicking human extracellular fluid composition, including ion concentrations and pH [15, 18, 19]. Ceftaroline (1/2 MIC) and carbapenems (at sub-MIC concentrations) were largely ineffective as monotherapies, although meropenem exhibited comparable activity to the combination therapy only under standard inoculum conditions in RPMI + 5%LB media. The combination of ceftaroline with either ertapenem or meropenem significantly enhanced bacterial killing compared with ceftaroline under both standard and high inoculum conditions irrespective of the media (Figure 1*A*). This effect was particularly pronounced in RPMI + 5%LB at high inoculum, where the combination showed superior activity compared with all monotherapies.

### In Vivo Murine Bacteremia Model of Ceftaroline-Resistant MRSA

Ceftaroline–carbapenem sensitization of ceftaroline-resistant MRSA to killing in vitro suggested potential in vivo utility, despite the lack of monotherapy activity. Although our murine model was relatively resistant to bacteremia infection by the ceftaroline-resistant MRSA, pilot studies revealed that a high bacterial inoculum (4.5 × 10^9^ CFU/mL) resulted in >50% mortality within 72 hours (data not shown). Using this challenge dose, mice were treated with subtherapeutic, humanized antibiotic regimens [16]. Notably, treatment with ceftaroline plus ertapenem resulted in a 1 log_10_ reduction in bacterial counts in the kidneys 24 hours after treatment, compared with ceftaroline alone (Figure 1*B*). No mice died or were excluded from analysis; data from 2 independent experimenters showed consistent results.

## DISCUSSION

As antibiotic resistance continues to rise, there is an urgent need for treatment strategies that not only address current resistance patterns but also anticipate and mitigate emerging threats. The present study evaluated one such strategy by examining a β-lactam–based combination therapy using ceftaroline plus carbapenems to counter ceftaroline resistance in MRSA. Our findings show that this combination significantly enhances bacterial killing in vitro. This effect persisted under high inoculum conditions typical of deep-seated infections and was even greater in physiologically modeled media. Synergy between ceftaroline and ertapenem was further validated in a murine bacteremia model using subtherapeutic, humanized dosing—demonstrating efficacy even at reduced drug exposures. Collectively, these results suggest that ceftaroline–carbapenem therapy may expand the therapeutic arsenal against refractory MRSA infections while building on prior mechanistic findings with implications for resistance suppression [11, 20].

Previous work from our group has demonstrated that this combination suppresses key resistance determinants, including *blaZ* and *PBP2a* [11]. This reduction highlights the ability of ceftaroline–carbapenem therapy to counteract adaptive bacterial responses, offering a mechanistic basis for its efficacy. In contrast, ceftaroline or carbapenem monotherapy upregulates β-lactam resistance genes (*blaR1*, *blaI*, *blaZ*, and *PBP2a*) in MRSA [11]. Exposure to carbapenems has also been associated with mutations in mecA (which encodes PBP2a), as well as additional mutations in PBP1 and PBP2—changes that may exacerbate ceftaroline resistance [11, 20]. While direct assessment of resistance emergence was not performed here, the combination's ability to suppress resistance genes [11] and retain activity against ceftaroline-resistant MRSA suggests it may mitigate some mechanisms of resistance development. Consistent with this, independent studies have demonstrated the efficacy of ceftaroline–meropenem combination therapy against ceftaroline-resistant MRSA in murine lung infection models [20], further supporting this approach in the management of resistant MRSA infections.

While not studied in this investigation, adjunctive carbapenems may also offer benefits through effects on immune responses and biofilm clearance [11, 21, 22]. Previous reports suggest carbapenems can enhance IL-1β–mediated host responses that are typically blunted in persistent *S. aureus* bacteremia [23, 24]. Such effects may further support their utility in recalcitrant infections, including those caused by ceftaroline-resistant MRSA.

Our study has several limitations. First, as a proof-of-concept study, it was conducted using a single ceftaroline-resistant MRSA strain, and further investigations are needed to validate these findings across diverse MRSA isolates and infection models. The relatively high inoculum required to establish infection in our murine bacteremia model may also reflect reduced virulence of this isolate. Moreover, the source and clinical origin of the CDC strain are unknown. Future studies should explore pharmacokinetically optimized regimens and assess host outcomes more comprehensively. Despite these limitations, our findings provide encouraging preliminary evidence for a potential strategy to counter ceftaroline-resistant MRSA and underscore the value of rationally designed combination therapies in overcoming antibiotic resistance.

## Supplementary Material

ofag009_Supplementary_Data

## Supplementary Data

Supplementary materials are available at *Open Forum Infectious Diseases* online. Consisting of data provided by the authors to benefit the reader, the posted materials are not copyedited and are the sole responsibility of the authors, so questions or comments should be addressed to the corresponding author.
