# Supplementary material for: Overcoming Ceftaroline Resistance in MRSA Using Ceftaroline–Carbapenem Combination Therapy
Source: Open Forum Infect Dis. 2026 Jan 20;13(1):ofag009. doi: 10.1093/ofid/ofag009 (PMC12818010; doi:10.1093/ofid/ofag009)
Supplement: ofag009_Supplementary_Data [file ofag009_supplementary_data.pdf]

Supplementary Table 1

Minimum inhibitory concentration (MIC) results and interpretation for methicillin-resistant *Staphylococcus aureus* AR-0703.

Drug	MIC (mg/L)	Interpretation
Cefoxitin	>16	Resistant
Ceftaroline	>4	Resistant
Clindamycin	>16	Resistant
Daptomycin	≤0.5	Susceptible
Doxycycline	8	Intermediate
Erythromycin	>8	Resistant
Gentamicin	>32	Resistant
Levofloxacin	8	Resistant
Linezolid	8	Resistant
Mupirocin	≤4	---
Oxacillin	>16	Resistant
Penicillin	>2	Resistant
Rifampin	>8	Resistant
Tedizolid	1	Intermediate
Tetracycline	>16	Resistant
Trimethoprim/sulfamethoxazole	>8	Resistant
Vancomycin	≤0.5	Susceptible

Adapted from the Centers for Disease Control and Prevention (CDC) and the Food and Drug Administration (FDA) antibiotic resistance isolate bank.
